# Anterior Cruciate Ligament Reconstruction with Internal Brace Augmentation Results in Fewer Reruptures Compared to Reconstruction without Augmentation

**DOI:** 10.1055/s-0044-1785663

**Published:** 2024-12-21

**Authors:** João Victor Novaretti, Claudio Paula Pessoa Dias Junior, Lucas Santos Lima, Joicemar Tarouco Amaro, Daniel Esperante Gomes, Moises Cohen

**Affiliations:** 1 Departamento de Ortopedia e Traumatologia, Centro de Ortopedia e Traumatologia do Esporte, Escola Paulista de Medicina, Universidade Federal de São Paulo, São Paulo, SP, Brasil; 2 Instituto Cohen, São Paulo, Brasil; 3 Departamento de Ortopedia e Traumatologia, Escola Paulista de Medicina, Universidade Federal de São Paulo, São Paulo, SP, Brasil

**Keywords:** anterior cruciate ligament, anterior cruciate ligament reconstruction, knee injuries, treatment outcome

## Abstract

**Objective** To compare the clinical outcomes of anterior cruciate ligament (ACL) reconstruction using autografts with and without internal brace augmentation.

**Methods** Data from patients who underwent ACL reconstruction with hamstring and quadriceps tendon autografts, with a minimum follow-up of one year, with or without internal brace augmentation were collected prospectively analyzed retrospectively. The Lysholm and Tegner functional scores were collected before and after surgery, as well as data on postoperative complications. For the comparison of means of the two groups, we used the Student *t* test or the Mann-Whitney non-parametric test, when the assumption of normality of the data was rejected.

**Results** In total, 55 patients underwent ACL reconstruction with internal brace augmentation and another 55 patients underwent ACL reconstruction without internal brace augmentation. The patients were aged between 16 and 63 years (mean of 32.7 ± 11.4 years). A total of 62 patients (56.4%) underwent ACL reconstruction with hamstring graft, and 19 patients (17.3%), with quadriceps tendon graft, with a diameter variation of 7 mm to 11 mm (mean of 8.95 ± 0.83 mm). The postoperative scores did not differ between the groups ( *p* > 0.05). Regarding the group submitted to ACL reconstruction with internal brace augmentation, 4 patients had complications: @ cases of arthrofibrosis, 2 (3.7%); 1 case of rerupture (1.8%); and 1 case of thrombosis (1.8%). In the group submitted to ACL reconstruction without augmentation, 7 patients manifested complications: 2 cases of arthrofibrosis (3.9%); 4 cases of rerupture (7.3%); and 1 case of infection (2%).

**Conclusion** The results of the present study show that fewer cases of ACL rerupture were observed after reconstruction with internal brace augmentation when compared with ACL reconstruction without augmentation, although no differences in functional scores were found.

## Introduction

Although several techniques already established for anterior cruciate ligament (ACL) reconstruction present excellent clinical and functional results, with a high rate of return to sport, 1 a rate of 1% to 11% of athletes undergo ACL rerupture. 2 Paterno et al. 3 observed that the risk of new injury to the same knee is approximately 15 times higher in athletes who have undergone ACL reconstruction and returned to sports after 1 year after surgery.

Several factors are associated with ACL rerupture, including early return to activities, errors in the surgical technique, biological graft failure, and traumatic re-rupture, which is the main reason and corresponds to more than half of the cases. 4 The onset of integration with the bone tunnels and graft vascularization occurs between 6 and 12 weeks after surgery, following the revascularization and remodeling process in an average time of 12 to 18 months. 5 During this period, aggressive rehabilitation or new trauma can lead to graft stretching or rupture. 6

New techniques have been developed in order to reduce the incidence of ACL re-ruptures. 7 8 The technique of ACL reconstruction with internal brace augmentation, using a high-strength polyethylene suture tape next to the graft, aims to protect the graft during its maturation phase, providing greater resistance to carry out the rehabilitation process while aiming at better functional results. 8 9 In addition, internal brace augmentation has been used for the medial collateral ligament in posteromedial corner repairs or reconstructions, 10 11 12 avulsion fractures of the posterior cruciate ligament, 13 repairs to the Achilles tendon, 14 reconstructions of lateral ankle instability, 15 and repair of the ulnar collateral ligament of the elbow with similar objectives. 16

The objective of the present study was to compare the clinical outcomes of patients undergoing ACL reconstruction with and without internal brace augmentation with functional scores and complications with at least one year of follow-up. We hypothesized that ACL reconstruction with internal brace augmentation would result in fewer reruptures and better functional scores when compared to ACL reconstruction without internal brace augmentation.

## Materials and Methods

**Fig. 1:**
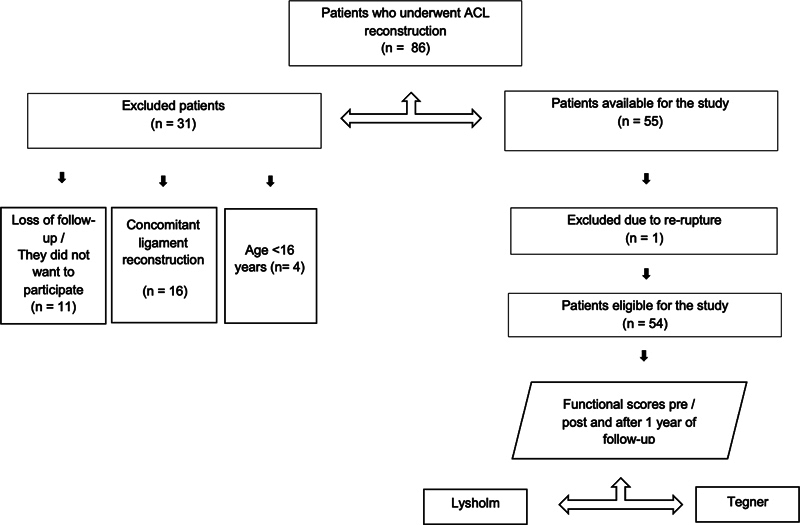
Patients who underwent anterior cruciate ligament (ACL) reconstruction with internal brace augmentation.

A review of the data prospectively collected was carried out after Institutional Review Board (IRB) approval was obtained (CAAE: 23241719.0.0000.0071). The present study included all patients aged over 16 years who underwent primary ACL reconstruction with autologous hamstring or quadriceps tendon grafts with and without internal brace augmentation at the 1-year follow-up ( Figs. 1 2 ). The patients were operated on by two fellowship-trained surgeons. The use of internal brace augmentation (FiberTape, Arthrex, Naples, FL, United States) was used in consecutive surgeries after the consecutive cases without internal brace augmentation in a non-randomized manner for the patients. The study excluded patients who refused to sign the free and informed consent form or who were lost to follow-up during this period; those who had a follow-up of less than one year; patients undergoing other concurrent ligament reconstructions or bone procedures; and those who underwent reconstruction with autologous graft from the patellar tendon, in cases of ACL reconstruction without internal brace augmentation. Patients undergoing treatment for meniscal injuries were not excluded. Age, sex, type and diameter of the graft, Lysholm and Tegner functional scores and postoperative complications were evaluated. The same ACL reconstruction technique was used by the two surgeons for ACL reconstructions with and without internal brace augmentation. 17

**Fig. 2:**
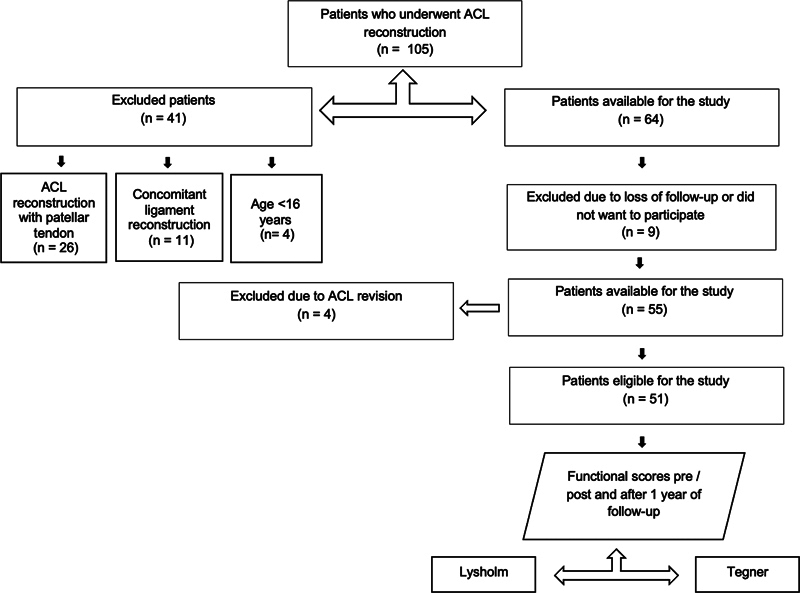
Patients who underwent ACL reconstruction without internal brace augmentation.

Additionally, both groups followed the same postoperative rehabilitation protocol. An a priori power analysis was used, with a minimum number of 42 cases from each group being demonstrated to assess rerupture in cases of ACL reconstruction with and without internal brace augmentation with a power of 80% and an alpha of 0.05. As noted, 32 patients were excluded from the study for various reasons. Rerupture cases were detected by physical examination and confirmed by magnetic resonance imaging (MRI). Patients who suffered rerupture did not complete the 1-year surgery questionnaires. The complications that occurred during this period were registered through data collected from medical records, obtained by another member of the study team.

## Surgical Technique

**Fig. 3:**
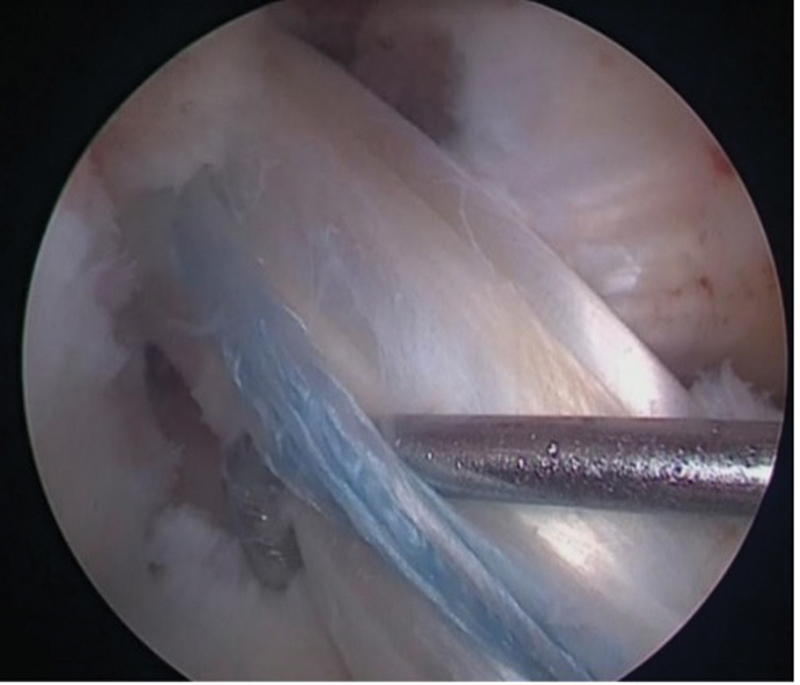
Probe located between the graft and the internal brace augmentation.

**Fig. 4:**
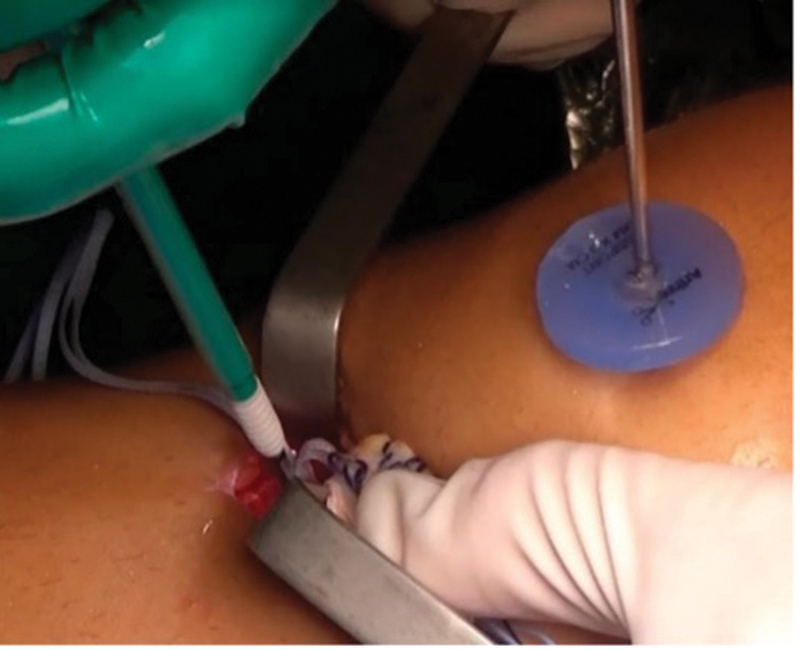
Independent fixation with internal brace augmentation with anchor in the tibia and presence in the probe maintained in its position shown in Fig. 3 .

Surgeries were performed using single-bundle technique and either hamstring or quadriceps tendon autograft. The tendons of the gracilis and semitendinosus were removed using a standard technique and prepared in a quadruple manner. 18 When a quadriceps tendon graft was chosen, it was removed without bone block, in its full thickness and by longitudinal incision. The tendons were fixed with an adjustable cortical button on the femur and with an absorbable interference screw on the tibia. In cases in which internal brace augmentation was used, a probe was placed ( Fig. 3 ) between the graft and internal brace augmentation to ensure that it was fixed with a slightly lower tension than that of the graft. Tibial fixation of the internal brace was performed with an anchor in the anterior cortex of the tibia, 1 cm distal from the lower edge of the tibial tunnel ( Fig. 4 ). Patients in both groups followed the same standard rehabilitation protocol for ACL reconstruction used in our institution.

## Statistical Analysis

Data distribution was analyzed using the Shapiro-Wilk test of normality. Normally distributed data were expressed as mean ± standard deviation values and comparisons were made using independent sample *t* -tests and repeated-measures analysis of variance (ANOVA) with a post-hoc Bonferroni correction. Non-parametric data were expressed as median and range values and compared using the Mann–Whitney U test. To test homogeneity between proportions, the Chi-square test or the Fisher exact test was used. Descriptive and comparative statistics were performed by a biostatistician using SPSS Statistics for Windows (SPSS Inc., Chicago Il., United States) software, version 17.0. Significance was set at *p* < 0.05.

## Results

Table 1 displays the characteristics of the included patients. Table 2 shows the complications in both groups. The group of ACL reconstruction with internal brace augmentation presented 1 rerupture (1.8%) while the group of ACL reconstruction without augmentation presented 4 reruptures (7.3%), despite not showing a statistically significant difference ( *p* = 0.363). The group of ACL reconstruction with internal brace augmentation had a greater graft diameter (9.10 ± 0.87 mm) than the group of ACL reconstruction without augmentation (8.82 ± 0.80 mm), despite not showing a statistically significant difference ( *p* = 0.092).

**Table 1:** Patient Data

	Anterior cruciate ligament reconstruction		
	Group with internal brace augmentation (n = 55)	Group without augmentation (n = 55)	*p*
Age (years): mean ± SD	32.7 ± 11.4	32.2 ± 10.6	0.806 ^*^
Sex: n (%)			0.294 ^**^
Female	20 (36.3)	15 (27.3)	
Male	35 (63.7)	40 (72.7)	
Side: n (%)			0.398 ^**^
Right	22 (40)	26 (47.2)	
Left	33 (60)	29 (52.7)	
Graft type: n (%)			0.242 ^**^
Hamstring	48 (87.3)	44 (80)	
Quadriceps	7 (12.7)	11 (20)	
Graft size: mean ± SD	9.10 ± 0.87	8.82 ± 0.80	0.092 ^*^

**Abbreviation:** SD, standard deviation.

Notes:^*^ Descriptive level of probability of the Student *t* -test; ^**^ descriptive level of probability of the Chi-squared test.

**Table 2:** Complications

	Anterior cruciate ligament reconstruction		
	Group with internal brace augmentation (n = 55): n (%)	Group without augmentation (n = 55): n (%)	*p* *
Complications	4 (7.27)	7 (12.72)	1.000
*Arthrofibrosis*	2 (3.63)	2 (3.63)	1.000
*Rerupture*	1 (1.81)	4 (7.27)	0.363
*Infection*	0 (0.0)	1 (1.81)	0.486
*Thrombosis*	1 (1.81)	0 (0.0)	1.000

Note:^*^ Descriptive level of probability of the Fisher exact test.

**Fig. 5:**
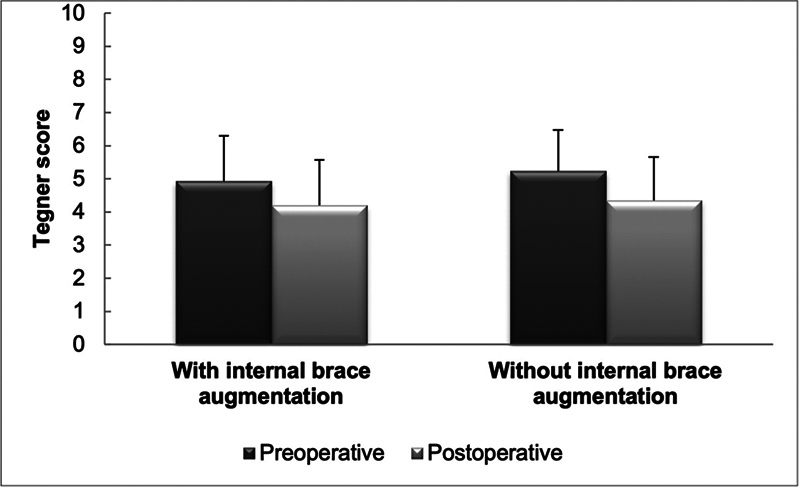
Tegner score before and after surgery.

A total of 105 patients completed Tegner and Lysholm scores at 1 year postoperatively, since 5 patients had ACL rerupture before this period. No significant difference was observed in the Tegner score preoperatively ( *p* = 0.41) and postoperatively ( *p* = 0.34) between the groups ( Fig. 5 ). There was a significant decrease in the Tegner score in the two groups after surgery ( *p* < 0.001).

**Fig. 6:**
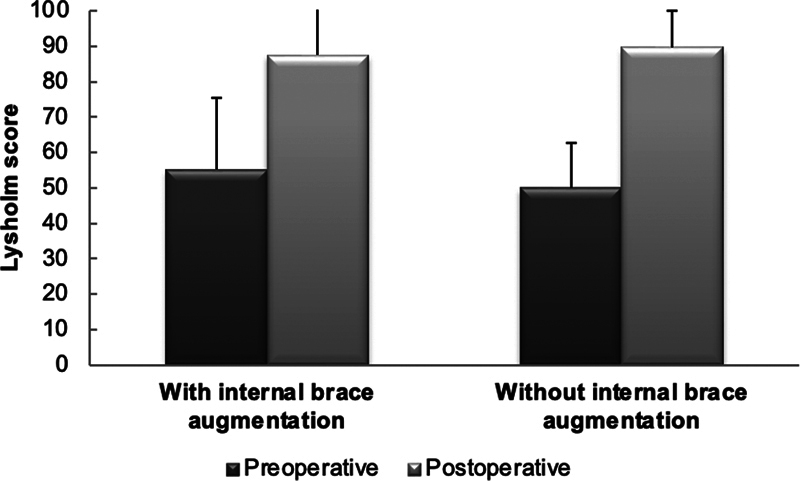
Lysholm score before and after surgery.

Regarding the Lysholm score, no significant difference was observed preoperatively ( *p* = 0.06) and postoperatively ( *p* = 0.51) between the groups ( Fig. 6 ). There was a significant increase in the Lysholm score in the two groups after surgery ( *p* < 0.001).

## Discussion

The main finding of the present study was that the rate of rerupture in the group of ACL reconstruction with internal brace augmentation was lower when compared with the group of ACL reconstruction without augmentation, despite not showing a statistically significant difference. The internal brace is fixed independently with less tension than the ACL graft, potentially functioning as a safety belt by dissipating some of the energy only when a higher load is imposed to the graft and, therefore, preventing graft rupture. 19 Hence, the use of internal brace augmentation might protect the ACL graft during the healing phase and enable early rehabilitation, which is in agreement with a recent biomechanical study 20 that showed that suture tape reinforcement of cadaveric ACL reconstruction significantly reduced graft elongation and failures.

Another advantage of using internal brace augmentation is the confidence in an accelerated rehabilitation program with early load bearing, with the device providing greater joint support. In this sense, internal brace augmentation acts as a secondary stabilizer after repair, which may enable accelerated rehabilitation and return to activities. 21 The ligament is encouraged to heal naturally, while not requiring any external braces. This enables accelerated rehabilitation with early mobilization, and the internal brace will theoretically protect against injury recurrence. As a result of this, rehabilitation following surgery can be approached differently from the one after standard ACL reconstruction. 22

A previous study 23 has shown a higher failure rate in revision ACL reconstruction with allograft reconstruction and internal brace augmentation. However, the previous literature 24 describes similar rates of failure between ACL reconstruction groups with and without internal brace augmentation. In addition, the use of internal brace is intended to reduce such complications. Although the addition of internal brace augmentation exposes the ACL to a new treatment with complications not yet well defined, its use has been studied in canines and prevented early failure. 25 Other devices have also been described in previous studies, 26 indicating a lower failure rate in ACL reconstruction.

The results of the present study demonstrate a significant improvement in the Lysholm score in the postoperative period, in comparison with the preoperative analysis of the two groups, but without differences between the groups. Additionally, a decrease in the Tegner score in the postoperative period was observed in comparison with the preoperative scores in both groups, with no differences between groups. This result may be explained by the one-year follow-up, since most patients have yet to return to sports at this time.

Regarding complications, the incidence of arthrofibrosis was equally described in the groups studied, corroborating the previous literature, 20 which reports that there is no difference in the use of the device. A study 25 on canine ACL reconstruction models with quadriceps tendon allografts with and without internal brace augmentation, showed no significant difference in constriction or stiffness six months postoperatively. However, despite being a rare complication, approximately 2% of patients present postoperative stiffness, which requires rapid intervention, aiming to prevent permanent arthrofibrosis, which can be presented in both groups. 26

There was a case reported in the present analysis with proven infection and described in the studied medical literature. Such a complication was reported in the group without internal brace augmentation, in which the infection rate was of 2.0%. Corroborating this data, the previous literature 27 shows that infection related to ACL reconstruction is a rare but potentially serious complication, with an incidence between 0.14% and 1.8%; however, it is worth noting that its occurrence is also associated with other factors, such as advanced age, the surgical technique adopted, surgeon's experience, preoperative preparation of the patient, among others.

Surgical procedures are associated with greater risks of deep venous thrombosis, although the recognition of risk factors, early mobilization, and ambulation are forms of preventive action. 28 One patient from the group with internal brace augmentation presented deep venous thrombosis, whose rate was of 1.8%, a value lower than the rate of 2.1% described in a meta-analysis in the literature. 29 However, although the patient in question underwent ACL reconstruction with internal brace augmentation, the use of the internal brace is unlikely to be related to higher rates of vascular complications, given that, according to a large cohort study 30 in which primary ACL reconstructions were analyzed, the authors found that 0.4% of the patients had deep vein thrombosis, and the only factor responsible for its occurrence was age ≥ 40 years.

The present study has limitations; one is that an objective assessment of patients was not carried out, since several patients were from other states and did not return for the one-year clinical evaluation. Due to the retrospective nature of the study, the patients were not randomized.

## Conclusion

The results of the present study show that fewer cases of ACL rerupture were observed after ACL reconstruction with internal brace augmentation, although no differences in functional scores were found when compared with ACL reconstruction without internal brace augmentation.
