# Supplementary material for: Anterior Cruciate Ligament Reconstruction with Internal Brace Augmentation Results in Fewer Reruptures Compared to Reconstruction without Augmentation
Source: Rev Bras Ortop (Sao Paulo). 2024 Dec 21;59(6):e868–75. doi: 10.1055/s-0044-1785663 (PMC11663066; doi:10.1055/s-0044-1785663)
Supplement: Related texts

# Research article: A reconstrução do ligamento cruzado anterior com aumento por cerclagem interna resulta em menos rerrupturas em comparação à reconstrução sem aumento

**Keywords:** ligamento cruzado anterior, reconstrução do ligamento cruzado anterior, resultado do tratamento, traumatismos do joelho

## Abstract

**Objetivo** Comparar os desfechos clínicos da reconstrução do ligamento cruzado anterior (LCA) usando autoenxertos com e sem aumento por cerclagem interna.

**Métodos** Os dados de pacientes submetidos à reconstrução do LCA com autoenxertos de tendões isquiotibiais e quadríceps e aumento por cerclagem interna ou não, além de acompanhamento mínimo de um ano, foram coletados de forma prospectiva e analisados retrospectivamente. Os escores funcionais de Lysholm e Tegner foram determinados antes e após a cirurgia, e dados sobre as complicações pós-operatórias também foram coletados. A comparação das médias dos dois grupos foi feita por meio do teste *t* de Student ou do teste não paramétrico de Mann-Whitney, em caso de rejeição da normalidade dos dados.

**Resultados** Ao todo, 55 pacientes foram submetidos à reconstrução do LCA com cerclagem interna, e outros 55 pacientes foram submetidos à reconstrução do LCA sem cerclagem interna. Os pacientes tinham entre 16 e 63 anos de idade (média de 32,7 ± 11,4 anos). No total, 62 pacientes (56,4%) foram submetidos à reconstrução do LCA com enxerto de isquiotibiais, e 19 pacientes (17,3%) receberam enxerto de tendão do quadríceps, com variação de diâmetro de 7 mm a 11 mm (média de 8,95 ± 0,83 mm). Os escores pós-operatórios não diferiram entre os grupos ( *p* > 0,05). Quatro pacientes do grupo reconstrução do LCA com cerclagem interna apresentaram complicações: 2 (3,7%) tiveram artrofibrose, 1 (1,8%) sofreu rerruptura, e 1 (1,8%), trombose. No grupo de reconstrução do LCA sem aumento, 7 pacientes manifestaram complicações: 2 (3,9%) tiveram artrofibrose, 4 (7,3%) sofreram rerruptura, e 1 (2%) apresentou infecção.

**Conclusão** Os resultados deste estudo mostram que houve menos casos de rerruptura do LCA após a reconstrução com aumento por cerclagem interna em comparação à reconstrução do LCA sem aumento, embora os escores funcionais não tenham apresentado diferenças.

## Introdução

Embora diversas técnicas já estabelecidas para a reconstrução do ligamento cruzado anterior (LCA) apresentem excelentes desfechos clínicos e funcionais, com alta taxa de retorno ao esporte, 1 1% a 11% dos atletas sofrem rerruptura do LCA. 2 Paterno et al. 3 observaram que o risco de nova lesão no mesmo joelho é aproximadamente quinze vezes maior em atletas submetidos à reconstrução do LCA e que retornaram ao esporte um ano após a cirurgia.

Diversos fatores estão associados à rerruptura do LCA, incluindo retorno precoce às atividades, erro na técnica cirúrgica, falha biológica do enxerto e rerruptura traumática, que é o principal motivo e corresponde a mais da metade dos casos. 4 O início da integração com os túneis ósseos e a vascularização do enxerto ocorrem de 6 a 12 semanas após a cirurgia, e acompanham o processo de revascularização e remodelamento em um tempo médio de 12 a 18 meses. 5 Nesse período, uma reabilitação agressiva ou um novo traumatismo pode levar ao estiramento ou à ruptura do enxerto. 6

Novas técnicas têm sido desenvolvidas com o objetivo de reduzir a incidência de rerrupturas do LCA. 7 8 A reconstrução do LCA com aumento por cerclagem interna, utilizando sutura de polietileno de alta resistência junto ao enxerto, tem como objetivo proteger o enxerto durante sua fase de maturação, ao proporcionar maior resistência para a realização do processo de reabilitação, visando melhores desfechos funcionais. 8 9 Além disso, o aumento por cerclagem interna tem sido utilizado no ligamento colateral medial em reparos ou reconstruções do canto posteromedial, 10 11 12 fraturas por avulsão do ligamento cruzado posterior, 13 reparos do tendão calcâneo, 14 reconstruções de instabilidade lateral do tornozelo 15 e reparo do ligamento colateral ulnar do cotovelo com objetivos semelhantes. 16

O objetivo deste estudo foi comparar os desfechos clínicos de pacientes submetidos à reconstrução do LCA com e sem cerclagem interna com escores funcionais e complicações por pelo menos um ano de acompanhamento. A hipótese foi a de que a reconstrução do LCA com aumento por cerclagem interna resultaria em menores taxas de rerruptura e melhores escores funcionais em comparação à reconstrução do LCA sem aumento por cerclagem interna.

## Materiais e Métodos

A revisão dos dados coletados de forma prospectiva foi realizada após a aprovação do Comitê de Ética Institucional (CAAE: 23241719.0.0000.0071). Este estudo incluiu todos os pacientes acima de 16 anos de idade submetidos à reconstrução primária do LCA com enxertos autólogos de tendão isquiotibial ou do quadríceps com e sem aumento por cerclagem interna e 1 ano de acompanhamento ( Figs. 1 2 ). Os pacientes foram operados por dois cirurgiões treinados. A cerclagem interna (FiberTape, Arthrex, Naples, FL, Estados Unidos) foi utilizada em cirurgias consecutivas após os casos consecutivos sem aumento por cerclagem interna, sem randomização dos pacientes. Os pacientes que se recusaram a assinar o termo de consentimento livre e esclarecido, aqueles perdidos durante o acompanhamento, com acompanhamento inferior a um ano, os submetidos a outras reconstruções ligamentares ou procedimentos ósseos concomitantes, ou submetidos à reconstrução com enxerto autólogo do tendão patelar nos casos de reconstrução do LCA sem aumento por cerclagem interna foram excluídos do estudo. Não foram excluídos pacientes em tratamento de lesões do menisco. Foram avaliados idade, sexo, tipo e diâmetro do enxerto, escores funcionais de Lysholm e de Tegner e complicações pós-operatórias. A mesma técnica de reconstrução do LCA foi utilizada pelos dois cirurgiões em procedimentos com e sem aumento por cerclagem interna. 17

**Fig. 1:** Pacientes submetidos à reconstrução do ligamento cruzado anterior (LCA) com aumento por cerclagem interna.

**Fig. 2:** Pacientes submetidos à reconstrução do ligamento cruzado anterior (LCA) sem aumento por cerclagem interna.

Além disso, ambos os grupos seguiram o mesmo protocolo de reabilitação pós-operatória. Uma análise de poder *a priori* demonstrou a necessidade de um número mínimo de 42 casos em cada grupo para a avaliação da rerruptura em casos de reconstrução do LCA com e sem aumento por cerclagem interna com poder de 80% e alfa de 0,05. Como observado, 32 pacientes foram excluídos do estudo por diversos motivos. Os casos de rerruptura foram detectados por exame físico e confirmados por ressonância magnética (RM). Os pacientes que sofreram rerruptura não preencheram os questionários de cirurgia de um ano. As complicações ocorridas nesse período foram registradas por meio de dados coletados em prontuários, obtidos por outro membro da equipe do estudo.

## Técnica Cirúrgica

As cirurgias foram realizadas pela técnica de feixe único e autoenxerto de tendão isquiotibial ou de quadríceps. Os tendões do grácil e semitendíneo foram removidos com técnica padrão e preparados de forma quádrupla. 18 O enxerto do tendão do quadríceps foi removido sem bloqueio ósseo, em toda a sua espessura e por incisão longitudinal. Os tendões foram fixados com botão cortical ajustável no fêmur e parafuso de interferência absorvível na tíbia. Nos casos com aumento por cerclagem interna, um probe foi colocado ( Fig. 3 ) entre o enxerto e a cerclagem para assegurar sua fixação com tensão ligeiramente menor do que a do enxerto. A fixação tibial da cerclagem interna foi realizada com âncora na cortical anterior da tíbia, 1 cm distal à borda inferior do túnel tibial ( Fig. 4 ). Os pacientes de ambos os grupos seguiram o mesmo protocolo padrão de reabilitação para reconstrução do LCA utilizado na instituição.

**Fig. 3:** Probe localizada entre o enxerto e o aumento por cerclagem interna.

**Fig. 4:** Fixação independente com aumento por cerclagem interna com âncora na tíbia e presença da probe mantida em sua posição mostrada na Fig. 3 .

## Análise Estatística

A distribuição dos dados foi analisada pelo teste de normalidade de Shapiro-Wilk. Os dados de distribuição normal foram apresentados por valores de média ± desvio padrão, e as comparações usaram testes *t* de amostras independentes e análise de variância ( *analysis of variance* , ANOVA, em inglês) de medidas repetidas com correção *post-hoc* de Bonferroni. Os dados não paramétricos foram apresentados por medianas e intervalos e comparados pelo teste U de Mann-Whitney. A homogeneidade entre as proporções foi determinada pelo teste do Qui-quadrado ou pelo teste exato de Fisher. As estatísticas descritivas e comparativas foram realizadas por um bioestatístico no programa SPSS Statistics for Windows (SPSS Inc., Chicago Il., Estados Unidos), versão 17.0. A significância foi estabelecida em *p* < 0,05.

## Resultados

A Tabela 1 mostra as características dos pacientes incluídos. A Tabela 2 mostra as complicações em ambos os grupos. O grupo de reconstrução do LCA com cerclagem interna teve 1 rerruptura (1,8%), enquanto o grupo de reconstrução do LCA sem aumento teve 4 rerrupturas (7,3%), mas não houve diferença estatisticamente significativa ( *p* = 0,363). No grupo reconstrução do LCA com cerclagem interna, o diâmetro do enxerto foi maior (9,10 ± 0,87 mm) do que no grupo de reconstrução do LCA sem aumento (8,82 ± 0,80 mm), mas sem diferença estatisticamente significativa ( *p* = 0,092).

**Tabela 1:** Dados dos pacientes

	Reconstrução do ligamento cruzado anterior		
	Grupo com aumento por cerclagem interna (n = 55)	Grupo sem aumento (n = 55)	*p*
Idade (anos): média ± DP	32,7 ± 11,4	32,2 ± 10,6	0,806*
Sexo: n (%)			0,294**
Feminino	20 (36,3)	15 (27,3)	
Masculino	35 (63,7)	40 (72,7)	
Lado: n (%)			0,398**
Direito	22 (40)	26 (47,2)	
Esquerdo	33 (60)	29 (52,7)	
Tipo de enxerto: n (%)			0,242**
Isquiotibial	48 (87,3)	44 (80)	
Quadríceps	7 (12,7)	11 (20)	
Tamanho do enxerto: média ± DP	9,10 ± 0,87	8,82 ± 0,80	0,092*

**Abreviatura:** DP, desvio padrão.

Notas:^*^ Nível descritivo de probabilidade do teste *t* de Student; ^**^ nível descritivo de probabilidade do teste do Qui-quadrado.

**Tabela 2:** Complicações

	Reconstrução do ligamento cruzado anterior		
	Grupo com aumento por cerclagem interna (n = 55): n (%)	Grupo sem aumento (n = 55) : n (%)	*p* *
Complicações	4 (7,27)	7 (12,72)	1,000
*Artrofibrose*	2 (3,63)	2 (3,63)	1,000
*Rerruptura*	1 (1,81)	4 (7,27)	0,363
*Infecção*	0 (0,0)	1 (1,81)	0,486
*Trombose*	1 (1,81)	0 (0,0)	1,000

Nota:^*^ Nível descritivo de probabilidade do teste exato de Fisher.

Ao todo, 105 pacientes responderam os escores de Tegner e Lysholm 1 ano após a cirurgia, uma vez que 5 pacientes tiveram rerruptura do LCA antes desse período. Não houve diferença significativa entre o escore de Tegner pré-operatório ( *p* = 0,41) e pós-operatório ( *p* = 0,34) entre os grupos ( Fig. 5 ). Houve diminuição significativa do escore de Tegner nos dois grupos após a cirurgia ( *p* < 0,001).

**Fig. 5:** Escore de Tegner antes e depois da cirurgia.

Em relação ao escore de Lysholm, não foi observada diferença significativa no pré-operatório ( *p* = 0,06) ou no pós-operatório ( *p* = 0,51) entre os grupos ( Fig. 6 ). Houve aumento significativo do escore de Lysholm nos dois grupos após a cirurgia ( *p* < 0,001).

**Fig. 6:** Escore de Lysholm antes e depois da cirurgia.

## Discussão

O principal achado deste estudo foi a menor taxa de rerruptura no grupo de reconstrução do LCA com aumento por cerclagem interna em comparação ao grupo sem aumento, apesar da ausência de diferença estatisticamente significativa. A cerclagem interna é fixada de forma independente e com menos tensão do que o enxerto do LCA, e talvez funcione como um cinto de segurança, ao dissipar parte da energia somente quando uma carga maior é imposta ao enxerto, o que, portanto, evita a sua ruptura. 19 Assim, o uso do aumento por cerclagem interna pode proteger o enxerto do LCA durante a fase de cicatrização e permitir a reabilitação precoce, o que é consistente com um estudo biomecânico recente 20 que mostrou que o reforço com cerclagem na reconstrução do LCA em cadáveres reduziu significativamente o alongamento e as falhas do enxerto.

Outra vantagem do uso do aumento por cerclagem interna é a confiança em um programa acelerado de reabilitação com suporte precoce de peso, pois a cerclagem proporciona maior suporte articular. Nesse sentido, o aumento por cerclagem interna atua como um estabilizador secundário após o reparo, o que pode acelerar a reabilitação e o retorno às atividades. 21 O ligamento é estimulado a cicatrizar naturalmente, sem necessidade de dispositivos externos. Isto permite uma reabilitação mais rápida com mobilização precoce, e a cerclagem interna, teoricamente, protege contra a recorrência da lesão. Como resultado disso, a reabilitação após a cirurgia pode ser abordada de forma diferente daquela após a reconstrução padrão do LCA. 22

Um estudo anterior 23 mostrou uma maior taxa de falha na reconstrução de revisão do LCA com reconstrução do aloenxerto e aumento por cerclagem interna. No entanto, a literatura anterior descreveu 24 taxas semelhantes de falha entre grupos de reconstrução do LCA com e sem aumento por cerclagem interna. Além disso, o uso da cerclagem interna destina-se a reduzir tais complicações. Embora o acréscimo da cerclagem interna exponha o LCA a um novo tratamento com complicações ainda não bem definidas, seu uso tem sido estudado em cães e preveniu falhas precoces. 25 Outros dispositivos também foram descritos na literatura anterior, 26 que indicou menor taxa de falhas na reconstrução do LCA.

Os resultados do presente estudo demonstram melhora significativa do escore pós-operatório de Lysholm em comparação à análise pré-operatória dos dois grupos, mas sem diferenças entre eles. Também foi observada diminuição do escore pós-operatório de Tegner em comparação aos escores pré-operatórios em ambos os grupos, sem diferenças entre eles. Esse resultado pode ser explicado pelo acompanhamento de um ano, uma vez que a maioria dos pacientes ainda não retornou à prática esportiva neste momento.

Quanto às complicações, a incidência de artrofibrose foi igualmente descrita nos grupos estudados, o que corrobora a literatura anterior, 20 que relatou não haver diferença no uso do dispositivo. Um estudo 25 de modelos caninos de reconstrução do LCA com aloenxertos de tendão do quadríceps, com e sem aumento por cerclagem interna, não mostrou diferença significativa na constrição ou rigidez seis meses após o procedimento. Porém, apesar de ser uma complicação rara, aproximadamente 2% dos pacientes apresentaram rigidez pós-operatória, o que requer intervenção rápida, visando prevenir artrofibrose permanente, que pode ocorrer em ambos os grupos. 26

Houve um caso relatado nesta análise com infecção comprovada e descrita na literatura médica estudada. Tal complicação foi relatada no grupo sem cerclagem interna, em que a taxa de infecção foi de 2,0%. Corroborando esses dados, a literatura anterior 27 mostrou que a infecção relacionada à reconstrução do LCA é uma complicação rara, mas potencialmente grave, com incidência entre 0,14% e 1,8%. Porém, vale ressaltar que sua ocorrência também está associada a outros fatores, como idade avançada, técnica cirúrgica adotada, experiência do cirurgião e preparo pré-cirúrgico do paciente.

Os procedimentos cirúrgicos estão associados a maiores riscos de trombose venosa profunda, embora o reconhecimento dos fatores de risco, a mobilização precoce e a deambulação sejam formas de atuação preventiva. 28 Um paciente do grupo com aumento por cerclagem interna apresentou trombose venosa profunda, cuja taxa foi de 1,8%, sendo inferior aos 2,1% descritos em uma metanálise. 29 Entretanto, embora o paciente em questão tenha sido submetido à reconstrução do LCA com aumento por cerclagem interna, é pouco provável que o uso da cerclagem esteja relacionado a maiores taxas de complicações vasculares, visto que um grande estudo de coorte 30 sobre reconstruções primárias do LCA constatou que 0,4% dos pacientes apresentavam trombose venosa profunda, e o único fator responsável pela sua ocorrência foi a idade igual ou superior a 40 anos.

Este estudo tem limitações; uma delas é a não realização de avaliação objetiva dos pacientes, pois vários pacientes eram de outros estados e não retornaram para a avaliação clínica de um ano. Devido à natureza retrospectiva deste estudo, os pacientes não foram randomizados.

## Conclusão

Os resultados deste estudo mostram que menos casos de rerruptura do LCA foram observados após a reconstrução do LCA com aumento por cerclagem interna, embora não tenha havido diferença nos escores funcionais em comparação à reconstrução do LCA sem aumento por cerclagem interna.
